# Comparing Gefitinib and Traditional Chemotherapy for Better Survival in Patients With Non-Small Cell Lung Cancer: A Systematic Review

**DOI:** 10.7759/cureus.33691

**Published:** 2023-01-12

**Authors:** Suthasenthuran Kanagalingam, Zargham Ul Haq, Nishok Victory Srinivasan, Aujala Irfan Khan, Ghadi D Mashat, Mohammad Hazique, Kokab Irfan Khan, Prasana Ramesh, Safeera Khan

**Affiliations:** 1 Internal Medicine, California Institute of Behavioral Neurosciences & Psychology, Fairfield, USA; 2 Medicine and Surgery, California Institute of Behavioral Neurosciences & Psychology, Fairfield, USA; 3 General Surgery, California Institute of Behavioral Neurosciences & Psychology, Fairfield, USA; 4 Research, California Institute of Behavioral Neurosciences & Psychology, Fairfield, USA; 5 Pediatrics, California Institute of Behavioral Neurosciences & Psychology, Fairfield, USA

**Keywords:** lung cancer, tyrosine kinase receptor inhibitors, epidermal growth factor receptor gene mutation, gefitinib, non-small cell lung carcinoma (nsclc)

## Abstract

Current non-small cell lung cancer (NSCLC) treatment consists of various combinations of surgery, chemotherapy, and/or radiation, depending on the tumor stage. Individuals with stage II-IIIa NSCLC undergo surgery, followed by combination chemotherapy containing cisplatin, such as vinorelbine + cisplatin. Epidermal growth factor receptor (EGFR) tyrosine kinase inhibitors (TKIs), such as gefitinib, act by inhibiting any signaling pathway containing the EGFR mutation and inhibiting the growth of NSCLC. TKI is a treatment option in advanced NSCLC, resulting in more prolonged progression-free survival (PFS). This manuscript aims to evaluate the influence of utilizing gefitinib - either alone or in combination with conventional chemotherapeutic drug regimens upon NSCLC patient profile survival parameters. A systematic literature review was conducted across multiple scientific literature repositories. The review was performed using the preferred reporting items for systematic reviews and meta-analyses (PRISMA) 2020.

There were six randomized clinical trials (RCT) and five retrospective studies. The overall consensus based on the end outcome of each published journal on the effectiveness of gefitinib as a treatment option for NSCLC indicated that there was a notable difference in overall survival (OS) and progression-free survival (PFS) and disease-free survival (DFS) datasets. Gefitinib use correlated with increased timeframes for multiple patient survival parameters within articles shortlisted in this investigation. However, more comprehensive investigations are required to validate such correlations. Gefitinib did demonstrate the potential to provide beneficial effects and counteract NSCLC within such patients.

## Introduction and background

Lung cancer is the leading cause of cancer deaths in the United States [1] and is subdivided into non-small cell lung cancer (NSCLC) and small cell lung cancer (SCLC). Common types of NSCLC include squamous cell carcinoma, adenocarcinoma, and large cell carcinoma [2]. NSCLC, as a class, is not as sensitive to chemotherapy and radiation as its counterpart SCLC [2]. Current NSCLC treatment consists of various combinations of surgery, chemotherapy, and/or radiation, depending on the tumor stage. Patients with stage II-IIIa NSCLC currently undergo surgery, followed by combination chemotherapy containing cisplatin, such as vinorelbine + cisplatin [3]. Patients in stage II-IIIa have a five-year overall survival (OS) rate between 36% and 49% [4]. 

Epidermal growth factor receptor (EGFR) is a transmembrane tyrosine kinase protein expressed in several normal neurogenic, mesenchymal, and epithelial tissues [5]. Tyrosine kinase (TK) is essential in regulating the signal pathway crucial for cellular function and survival [6]. Furthermore, within several advanced NSCLC tumors, mutations in EGFR can be found, allowing for uncontrolled cellular proliferation. EGFR tyrosine kinase inhibitors (TKIs), such as Gefitinib (G), inhibit the signaling pathway containing the EGFR mutation and NSCLC growth [6]. TKI is a treatment option in advanced NSCLC, resulting in longer progression-free survival (PFS) [3]. 

Recently, ADJUVANT-CTONG1104, a randomized phase three trial, indicated increased disease-free survival (DFS) with standard vinorelbine + cisplatin + G of 28.7 months, compared to 18.0 months on standard therapy without G [7]. In a separate clinical trial phase, two EVAN studies, patients in stage IIIa were treated adjuvant with erlotinib (EGFR inhibitor), leading to an improved two-year DFS when compared with adjuvant chemotherapy vinorelbine + cisplatin [8]. However, TKI treatment develops resistance following approximately eight to 12 months [9]. The resistance mechanism is possible due to: (a) parallel signaling pathway activation, (b) downstream activation of the signaling pathway, (c) secondary EGFR mutation, and/or (d) histological transformation [9]. One solution to such acquired chemoresistance is combining TKI with cytotoxic chemotherapy, allowing this treatment to induce apoptosis and suppress protein kinase B (Akt) synergistically [10]. 

Despite NSCLC therapeutic advancements with TKI, G has not yet reached the decisive level of a comparable TKI such as Imatinib. Imatinib is typically employed in chronic myelogenous leukemia (CML), allowing it to be treated as a chronic disease [10]. In this systematic review, we will compare the use of gefitinib as an alternative treatment in NSCLC and compare it to traditional chemotherapies. 

## Review

Methods

The systematic review was conducted using the preferred reporting items for systematic reviews and meta-analyses (PRISMA) 2020 [11]. 

Search Source and Strategy 

Initial searches were performed through the following databases: PubMed, PubMed Central (PMC), Medline, and Cochrane Library. Keywords used in the search were Non-Small Cell Lung Cancer, Chemotherapy, and Gefitinib. Keywords were used with the Boolean "AND" to obtain results. Medical subject heading (MeSH) search strategy was also used where applicable: (1) NSCLC - ("Carcinoma, Non-Small-Cell Lung/drug therapy"[Mesh] OR "Carcinoma, Non-Small-Cell Lung/radiotherapy"[Mesh] OR "Carcinoma, Non-Small-Cell Lung/surgery"[Mesh] OR "Carcinoma, Non-Small-Cell Lung/therapy"[Mesh]), and (2) Gefitinib - ( "Gefitinib/administration and dosage"[Majr] OR "Gefitinib/adverse effects"[Majr] OR "Gefitinib/therapeutic use"[Majr] OR "Gefitinib/toxicity"[Majr]). 

Screening and Eligibility 

Inclusion criteria were (a) articles published in the English language, (b) adult population, (c) articles relevant to the research question, (d) published in the last five years, and (e) full-text articles. Exclusion criteria were (a) grey literature and (b) unpublished literature, and (c) pediatric population. Duplicates were removed, followed by initial screening based on title and abstracts. The quality of each article was analyzed and further filtered using the following risk bias assessments: Cochrane risk bias assessment tool and Joanna Briggs Institute (JBI) critical appraisal checklist (Table 1). 

**Table 1:** Quality appraisal tools employed for this study JBI: Joanna Briggs Institute, RCT: Randomized clinical trial

Study Type	Quality Appraisal Tool
RCT	Cochrane Bias Assessment Tool [12]
Retrospective Study	JBI Checklist [13]

Results

Search Results 

**Figure 1:**
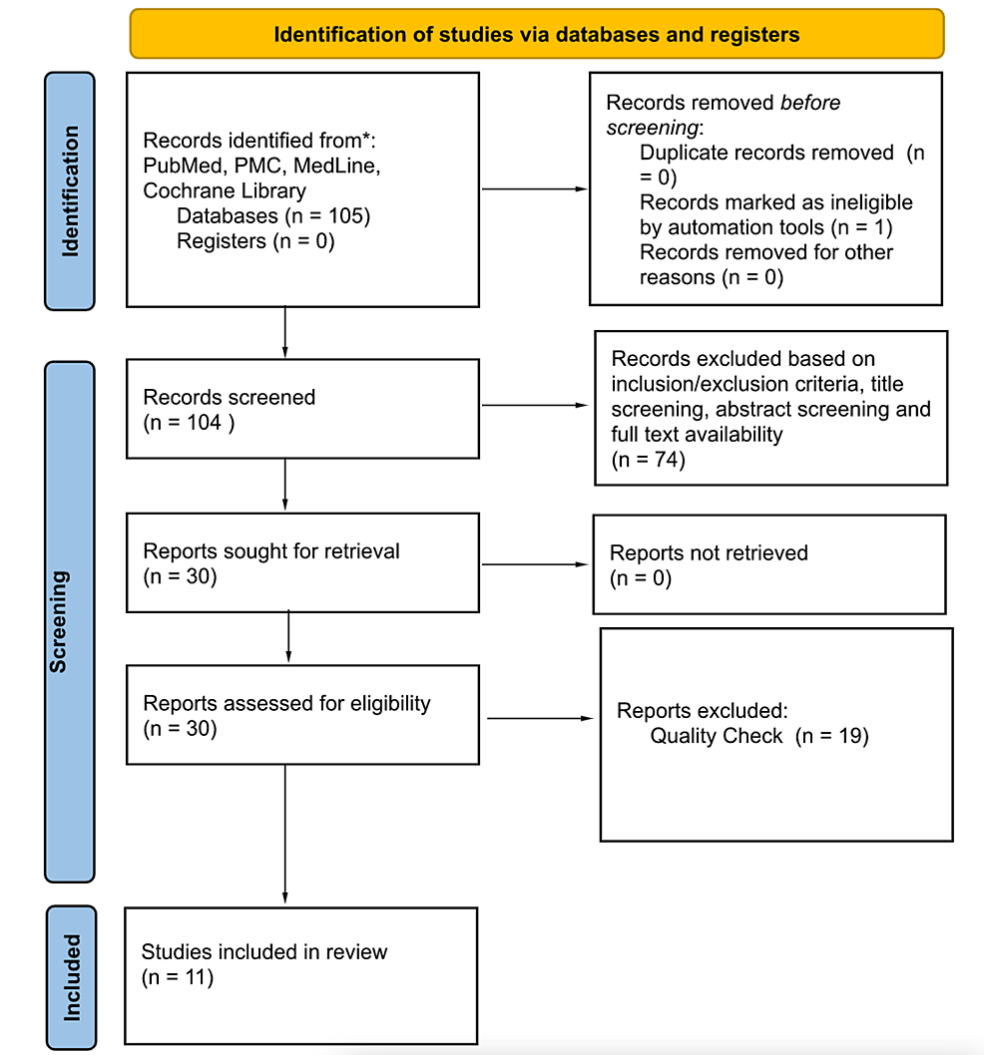
Preferred reporting items for systematic reviews and meta-analyses (PRISMA) 2020 PMC: PubMed central

Database: PubMed, PubMed Central (PMC), Medline, and Cochrane Library searches yielded 105 published articles. After the removal of the duplicates and articles filtered based on the inclusion and exclusion criteria, a total of 30 articles were left. The screening and eligibility process narrowed it down to 11 relevant journal articles (Table 1 and Figure 1). There were six randomized clinical trials (RCT) and five retrospective studies.

Article Results 

A total of 11 published journal articles that had gone through extensive selection analysis resulted in a total number of patients of 1,958 (Table 2). The overall consensus based on the end outcome of each published journal on the effectiveness of Gefitinib (G) as a treatment option for non-small cell lung cancer (NSCLC) indicated a notable difference in overall survival (OS), progression-free survival (PFS), and/or disease-free survival (DFS). 

**Table 2:** Publication summary of the purpose, number of patients, study type, and conclusion NSCLC: Non-small cell lung cancer, G: Gefitinib, CT: Chemotherapy, EGFR: Epidermal growth factor receptor, TKI: Tyrosine kinase inhibitors, RCT: Randomized clinical trial, DFS: Disease-free survival, OS: Overall survival, PFS: Progression-free survival, TAI: Transarterial infusion, BSC: Best supportive care, VP: Vinorelbine plus cisplatin, CP: Carboplatin and pemetrexed, CD: Cisplatin plus docetaxel

Author and Year of Publication	Purpose	Number of Patients	Study Type	Conclusion
Zhong et al. 2021 [3]	Randomized phase three trial on patients with epidermal growth factor (EGFR) mutation stage II-IIIA non-small cell lung cancer (NSCLC) gave adjuvant Gefitinib (G) treatment versus vinorelbine plus cisplatin (VP) to assess overall survival (OS).	222	RCT	G Improved disease-free survival (DFS) over standard care chemotherapy. However, DFS did not indicate a significant OS difference. But it was indicated the OS for the G group was the longest observed in this patient group compared to historical data.
Noronha et al. 2020 [10]	Randomized phase three open-label trial with advanced NSCLC patients with EGFR mutation. The study added carboplatin and pemetrexed (CP) chemotherapy to G versus G alone to evaluate for improved outcomes.	350	RCT	The addition of pemetrexed and carboplatin chemotherapy to G increased OS and PFS. However, the treatment also increased toxicity.
Hosomi et al. 2020 [14]	A randomized clinical trial of EGFR tyrosine kinase inhibitors (TKI) G combined with chemotherapy carboplatin plus pemetrexed. The trial evaluated the efficacy and safety of the combination.	345	RCT	G combined with CP improved progression-free survival (PFS). The OS needs further validation. Acceptable toxicity profile, but OS benefit will require further testing.
Tada et al. 2022 [15]	The efficacy of G was investigated as adjuvant therapy. Randomized phase three study with patients with stage II-III NSCLC with EGFR mutation were enrolled. G versus VP.	234	RCT	G prevented early relapse but did not prolong OS or DFS.
Jian et al., 2017 [16]	The study focused on the combination treatment of G plus Gemcitabine plus Carboplatin versus Gemcitabine plus Carboplatin in stage IIIB/IV non-squamous NSCLC.	219	RCT	OS was longer in the group with the combination of G plus Gemcitabine plus Carboplatin.
Yoshioka et al., 2019 [17]	Phase three studies were done to compare the safety and efficacy of G versus Cisplatin plus Docetaxel (CD). Patients with stage IIIB/IV or postoperative recurrent EGFR mutation NSCLC.	172	RCT	G did not result in OS benefits over CD, as first-line treatment may be due to a high cross-over rate.
Hirsch et al. 2018 [18]	Retrospective study of long-term (>10 years), tolerability, safety, and survival of patients on G with advanced NSCLC.	79	Retrospective Study	G resulted in an excellent long-term safety profile. G was well tolerated.
Zhang et al. 2019 [19]	A retrospective study on the safety and benefit of G plus transarterial infusion (TAI) versus G alone. Patients with >7 cm NSCLC with EGFR mutation.	92	Retrospective Study	Combination therapy of G plus TAI was tolerated well and possibly improved tumor reduction and PFS.
Kashiwabara et al. 2020 [20]	A retrospective study was done on the survival benefit of G in the super elderly (age ≥ 85 years). Patients that received the best supportive care alone (BSC), cytotoxic chemotherapy (CT), or EGFR-TKI were compared.	69	Retrospective Study	G was useful as salvage therapy in patients with NSCLC with active EGFR mutation.
Choi et al. 2018 [21]	Retrospective study of EGFR Exon 19 deletion and benefit of G usage as the first line. The study focused on the variable mutations - exon 19 deletion, L858R mutation, and dual or uncommon mutation.	60	Retrospective Study	EGFR mutation in exon 19 had favorable PFS and OS in patients treated with G as the first line.
Xie et al. 2018 [22]	A retrospective study on NSCLC was completely resected with stage II-IIIA EGFR mutation (exon 19 deletion or exon 21 Leu858Arg). Compared standard chemotherapy versus G.	116	Retrospective Study	G was superior to standard chemotherapy in NSCLC. DFS was higher in the G group and reduced toxicity in completely resected stage II-III EGFR mutation patients.

**Discussion** 

Overall Findings of the Selected Research Papers

The composition of the 11 research articles that were shortlisted following this comprehensive literature review comprised four articles where gefitinib (G) was employed in combination therapies for non-small cell lung cancer (NSCLC) [10,14,16,19], with the remainder of the articles focusing on gefitinib as replacement therapy for conventional chemotherapeutic strategies [3,15,17,18,20-22]. In terms of survival parameter effects, such as overall survival (OS), disease-free survival (DFS), and progression-free survival (PFS), the study by Zhong and colleagues demonstrated that gefitinib improved DFS over standard-care chemotherapy [3]. However, DFS did not indicate a significant variation in OS within this study, although the OS dataset within the gefitinib group was the most prolonged compared to historical data [3]. In comparison, three other studies confirmed that gefitinib (either alone or in combination) could extend OS periods within patient cohorts exposed to the drug [10,16,21]. Furthermore, gefitinib (either alone or in combination) was found to have beneficial effects in extending PFS in four randomized controlled/retrospective studies [10,14,19,21]. Overall, the DFS was extended by gefitinib (either alone or in combination) across a total of two articles within this shortlist [3,22]. Since none of the 11 shortlisted articles focused on all three survival parameters simultaneously within their investigations, a distinct outcome cannot be concluded, although there is a correlation between gefitinib use and survival parameter extensions observed from such studies.

Regarding gefitinib-induced toxicity within patients and other safety profile parameters, three articles found that gefitinib reduced toxicity levels within patient cohorts during such individual investigations [18,19,22]. In comparison, Noronha and colleagues (2020) study found that a combination therapy consisting of gefitinib/carboplatin/pemetrexed did exacerbate toxicity levels within study cohorts. However, combinatory therapy was effective in prolonging patient survival odds [10]. Overall, the trend across the 11 shortlisted articles indicated that gefitinib did not affect/was beneficial upon overall therapy-based toxicity within study cohorts.

Gefitinib Overall Effectiveness as a Replacement Therapy

Upon qualitative comparative analyses of the four shortlisted studies that employed gefitinib as a replacement NSCLC therapy for conventional chemotherapeutic options, two studies reported an increased DFS period within study cohorts exposed to gefitinib [3,22]. In addition, only one article reported an increase in the PFS period following gefitinib replacement therapy [21]. Consequently, distinct inferences on gefitinib sole therapies over survival parameter prolonging in NSCLC cohorts remain debatable. Furthermore, toxicity levels were identified within two separate studies using gefitinib replacement treatment regimens [18,19,22]. Again, a distinct inference of gefitinib-alone therapy over toxicity levels cannot be defined since the remainder of the articles within the identified shortlist either did not report any toxicity variations in such patient cohorts or did not investigate such issues.

Gefitinib Overall Effectiveness as a Combinatory Therapy

Upon qualitative comparative analyses of the four shortlisted studies that employed gefitinib as part of combination therapy, only two articles did observe increased OS periods within patient cohorts [10,16]. In contrast, most such studies did observe PFS prolonging for study cohorts, suggesting that gefitinib-based combination therapies (either with trans-arterial infusions or specifically carboplatin/pemetrexed) can extend PFS in NSCLC patients [10,14,19,21]. However, data regarding gefitinib-based combination studies on toxicity levels remains unclear, as individual studies reported varying observations on this issue.

Gefitinib Overall Safety Profile/Quality-of-Life Extension

One of the most prevalent inferences gathered across all investigated studies was that gefitinib was highly effective in extending disease-free survival (DFS) within patient cohorts compared to conventional chemotherapeutic measures [3,22]. Gefitinib was also highlighted to have a beneficial effect by extending overall survival (OS) timeframes in patients - either alone or in combination with other chemotherapeutic agents such as pemetrexed/carboplatin and gemcitabine/carboplatin [3,10,16,21]. Case in point, in the study conducted by Zhong and colleagues alone, OS was extended by over 50% when using gefitinib instead of vinorelbine/cisplatin (VP) treatment (75.5 and 62.8 months, respectively; HR 0.92; 95% CI, 0.62 to 1.36; P = .674), while three-year DFS rates were 39.6% and 32. 5% with gefitinib and VP (P = .316), respectively [3]. However, this was not identified across all selected articles shortlisted within this systematic literature review.

In addition, this was also not identified in another recently published investigation. The systematic review and network meta-analysis conducted by Chan and colleagues in 2022 focused on the potential beneficial effects of first-line therapies on OS timeframes within advanced epidermal growth factor receptor (EGFR) mutated NSCLC Asian patient cohorts carrying the L858R mutation [23]. Overall, this comprehensive investigation probed 18 study trials across 1852 Asian NSCLC clinical cases and 12 differing NSCLC therapies, including a range of EGFR tyrosine kinase inhibitors (TKIs) such as gefitinib, together with EGFR- TKI/other chemotherapeutic agent combinatory therapies, such as gefitinib/lapatinib and gefitinib/pemetrexed treatment regimens [23]. This particular systematic review and network meta-analysis revealed that Asian cases of NSCLC carrying the L858R mutation had no beneficial effects concerning OS timeframe extensions following any such therapy used across the 18 trials [23]. However, Gefitinib plus pemetrexed-linked chemotherapy, dacomitinib, and erlotinib plus bevacizumab had enhanced rankings - with p scores of 89%, 82%, and 68%, respectively - proving to be efficient in extending PFS timeframes within such clinical cases post-treatment, even though this also led to an increased incidence rate of grade 3 (or higher) adverse conditions within such patient cohorts [23].

However, the recent retrospective cohort investigation carried out by Dai and colleagues in 2022 that solely focused on the influence of gefitinib when administered in combination with conventional chemotherapeutic agents in advanced NSCLC corroborated our study results [24]. This particular investigation analyzed therapeutic outcomes within a total of 120 clinical cases of advanced EGFR mutation-positive NSCLC across two separate cohorts control cohort (CC) - treated with conventional chemotherapy alone; observation cohort (OC) - treated with conventional chemotherapy + gefitinib [24]. The dataset outcomes from this specific investigation indicated that median values for PFS and OS were both extended within the observation cohort in comparison to the control cohort (PFS: eight months in OC versus five months in CC; OS: 24.0 months in OC versus 18.0 months in CC, respectively) [24]. Such study results further confirm that the introduction of gefitinib, in tandem with conventional chemotherapeutic options, carries significant clinical value in extending PFS and OS statistics within such NSCLC cases.

Furthermore, other studies - listed in our review shortlist - also demonstrated that progression-free survival timeframes were positively affected by gefitinib therapy, either as a standalone treatment or in combination with carboplatin pemetrexed/carboplatin therapies or together with trans-arterial infusion therapy [10,14,19,21]. Case in point, in the study by Hosomi and colleagues alone, the combined-drug cohort exhibited enhanced objective response rates (ORR) and PFS than the gefitinib cohort individually (ORR, 84% v 67% [P < .001]; PFS, 20.9 v 11.9 months; hazard ratio for death or disease progression, 0.490 [P < .001]) [14]. Gefitinib was also proven effective in circumventing early relapse episodes within Stage II-III NSCLC patients, increasing five-year OS rates by 3.4% [15]. Furthermore, gefitinib was identified to be adequately tolerated by patients, with a promising long-term safety profile. However, this was not fully corroborated when used in combination with pemetrexed/carboplatin therapy [10,14,18,22]. Interestingly, the recent network meta-analysis by Haeussler and colleagues in 2022 assessed the comparative effectiveness and safety profiles for multiple first-line treatment options indicated for advanced EGFR mutation-positive NSCLC clinical cases [25]. This investigation performed a Bayesian network meta-analysis across multiple first-line treatments in such clinical cases, with gefitinib/erlotinib being the baseline reference therapy for all comparative analyses [25]. This study's dataset outcomes highlighted that other therapeutic combination, such as ramucirumab/erlotinib, was more effective and had an enhanced safety profile compared to the gefitinib/erlotinib therapeutic regime [25].

Gefitinib Effectiveness on EGFR Mutations

Regarding the effectiveness of gefitinib within NSCLC patients having an EGFR-mutated status, gefitinib was found to have elevated efficacy levels in salvage therapies within NSCLC patients > 85 years of age, extending PFS by 1.4 months (p = 0.070) [20]. Gefitinib was also highly effective within NSCLC patients carrying the exon 19 deletion mutation and/or exon 21 Leu858Arg mutation status [3,19-22]. Typically, NSCLC patients did not receive an EGFR-TKI along with chemotherapy, though selected studies were conducted to probe such a therapeutic combination. Noronha and colleagues' 2020 randomized phase three open-label trial consisted of 350 patients with NSCLC + EGFR mutation treated with either gefitinib or gefitinib and carboplatin combination therapy. The median PFS outcomes for gefitinib/carboplatin combination therapy and gefitinib alone were 16 months (95% confidence interval (CI), 13.5 to 18.5 months) and eight months (95% CI, seven to nine months), respectively [10]. Interestingly, the 2021 study by Zhong and colleagues consisted of a randomized phase two trial (n=222) with EGFR mutation-positive NSCLC with resected stage II-IIIA to evaluate the effects of G treatment in comparison to vinorelbine plus cisplatin (VP). Median OS 75.5 (95% CI, 46.6 to not calculable (NC)) and 62.8 months (95% CI 45.8 NC) with G and VP, respectively [3]. The OS did not result in a significant difference between G and VP. However, approximately 48% of patients that obtained adjuvant chemotherapy had EGFR mutation but could not get EGFR-TKIs because of the cost associated with the drug in China, as most patients had to pay out of pocket, in turn limiting its use [3].

This review study has limitations, mainly stemming from the non-inclusion of specific meta-analyses, such as the employment of the random-effects model, determination of effect estimates, and confidence intervals/statistical/design evaluation for heterogeneity sub-group sensitivity evaluations, and small-investigation effects [26]. In addition, the possibility of employing network-based meta-analyses should be considered for future similar studies to enhance the proper and accurate evaluation of relative efficacy levels for multiple therapeutic strategies adopted over a spectrum of randomized controlled trials, with consequent robust data collection from such studies [26]. Such Bayesian network evaluations can maximize estimate precision levels (in comparison to the sole and direct scientific data), together with enabling the evaluation of comparative effectiveness for two specific therapies, even when no investigation previously conducted a direct comparative analysis [26]. Furthermore, this review did not include the emerging evidence regarding gefitinib resistance in NSCLC and additional novel therapeutic options that are currently being developed to mitigate such chemotherapeutic resistance issues within clinical cases of EGFR mutation-positive NSCLC, including the possible deployment of long non-coding ribonucleic acid (RNA) and microRNA molecular players to carry out such chemoresistance mitigation effector functions [27-29].

## Conclusions

Gefitinib did demonstrate the potential to provide beneficial effects and counteract non-small cell lung cancer (NSCLC) in patients. Such a trend was observed when gefitinib was employed as replacement therapy for conventional chemotherapy and combined with conventional chemotherapeutic drug regimens such as pemetrexed/carboplatin and gemcitabine/carboplatin. However, this was not identified across all selected articles shortlisted within this systematic literature review. Regarding the effectiveness of gefitinib within NSCLC patients having an epidermal growth factor receptor (EGFR) mutated status, gefitinib was found to have elevated efficacy levels in salvage and within NSCLC patients carrying the exon 19 deletion mutation and/or exon 21 Leu858Arg mutation status.

In essence, gefitinib use correlated with increased timeframes for multiple patient survival parameters within articles shortlisted in this investigation. However, more comprehensive investigations are required to validate such correlations. Once such potential is consolidated and existing gefitinib chemoresistance issues circumvented, gefitinib can be widely deployed as a novel and low-risk biologic-based chemotherapeutic agent against NSCLC.
